# The Proactive Synergy Between Action Observation and Execution in the Acquisition of New Motor Skills

**DOI:** 10.3389/fnhum.2022.793849

**Published:** 2022-03-24

**Authors:** Maria Chiara Bazzini, Arturo Nuara, Emilia Scalona, Doriana De Marco, Giacomo Rizzolatti, Pietro Avanzini, Maddalena Fabbri-Destro

**Affiliations:** ^1^Consiglio Nazionale delle Ricerche, Istituto di Neuroscienze, Parma, Italy; ^2^Dipartimento di Medicina e Chirurgia, Università degli Studi di Parma, Parma, Italy; ^3^Istituto Clinico Humanitas, Humanitas Clinical and Research Center, Milan, Italy

**Keywords:** mirror neurons, mirror mechanisms, sports training, motor control, action observation treatment (AOT), imitation

## Abstract

Motor learning can be defined as a process that leads to relatively permanent changes in motor behavior through repeated interactions with the environment. Different strategies can be adopted to achieve motor learning: movements can be overtly practiced leading to an amelioration of motor performance; alternatively, covert strategies (e.g., action observation) can promote neuroplastic changes in the motor system even in the absence of real movement execution. However, whether a training regularly alternating action observation and execution (i.e., Action Observation Training, AOT) may surpass the pure motor practice (MP) and observational learning (OL) remains to be established. To address this issue, we enrolled 54 subjects requiring them to learn tying nautical knots via one out of three types of training (AOT, MP, OL) with the scope to investigate which element mostly contributes to motor learning. We evaluated the overall improvement of each group, along with the predictive role that neuropsychological indexes exert on each treatment outcome. The AOT group exhibited the highest performance improvement (42%), indicating that the regular alternation between observation and execution biases participants toward a better performance. The reiteration of this sequence provides an incremental, adjunct value that super-adds onto the efficacy of motor practice or observational learning in isolation (42% > 25% + 10%, i.e., OL + MP). These findings extend the use of the AOT from clinical and rehabilitative contexts to daily routines requiring the learning and perfectioning of new motor skills such as sports training, music, and occupational activities requiring fine motor control.

## Introduction

Motor learning can be defined as a process that leads to relatively permanent changes in the own motor behavior through repeated interactions with the environment (Wolpert et al., 2001; Halsband and Lange, 2006; Krakauer et al., 2019). This process allows acquiring new motor skills and enriching the motor repertoire (Schmidt and Lee, 2005; Wulf et al., 2010; Gatti et al., 2013). Motor learning covers different roles during the whole life according to the life stages. During childhood, motor learning allows the child to explore and interact proficiently with the surrounding environment (Lubans et al., 2010; Serrien et al., 2014; Adolph and Hoch, 2019). During adulthood, people use motor learning to get specialized in motor abilities required to carry out professional (Wong and Matsumoto, 2008; Khan et al., 2020) or entertainment tasks like sports (Yarrow et al., 2009) and music (Altenmueller and McPherson, 2008); finally, during elderly age, motor learning is still fundamental to make people adapt to their time-changing bodies and physical abilities (Nunes et al., 2014).

An extensive literature has documented that repeated stimulations of the motor system are necessary to instantiate motor learning (Willingham, 1998; Krakauer et al., 2019). The most obvious strategy is the repeated voluntary physical training characterized by an initial phase of trial-and-error practice necessary to reach an appropriate movement pattern and an intensive physical repetition phase to consolidate the correct motor strategy (Magill, 2000; Coker, 2004). Motor practice efficacy manifests in reduced movement duration and errors and increased accuracy during task execution (Moore and Marteniuk, 1986; Dugas and Marteniuk, 1989). The learning of new motor skills is associated with the reorganization of an extensive motor network (Chang, 2014), encompassing at the cortical level the premotor and primary motor cortices, the primary somatosensory cortex and the superior parietal lobule and, at the subcortical level, the thalamus, basal ganglia, and cerebellum (Hardwick et al., 2013).

Most of these neural substrates, especially at the cortical level, can also be activated by covert motor strategies that do not require a motor task execution. Among them, it is worth mentioning the case of action observation and motor imagery, both capable of eliciting the activation of frontal and parietal areas shared with action execution (Hardwick et al., 2018). When administered for training purposes, both action observation (Stefan et al., 2005; Celnik et al., 2008) and motor imagery (Cicinelli et al., 2006; Ruffino et al., 2019) induce neuroplasticity processes reflecting in long-lasting changes in corticomotor excitability. These latter are acknowledged as the major neurobiological substrates of behavioral learning outcomes (Sale et al., 2017). This notion explains why motor learning can also be achieved through the repeated observation of an action (i.e., observational learning) (Horn and Williams, 2004; Ashford et al., 2006; Vogt and Thomaschke, 2007; Maslovat et al., 2010; Ste-Marie et al., 2012; Hodges, 2017; for reviews) or its motor imagery (Pascual-Leone et al., 1995; Dickstein and Deutsch, 2007; Gentili et al., 2010).

Beyond pure models of training (motor practice, observational learning, and motor imagery), a mixed model has been recently proposed, called Action Observation Training (AOT). AOT inherently alternates action observation and execution, thus potentially combining the reciprocal advantages of motor practice and observational learning. Its efficacy in promoting functional recovery was reported in several clinical conditions such as stroke, Parkinson’s disease, cerebral palsy, multiple sclerosis, and post-surgical orthopedics [see for an extensive review, Rizzolatti et al. (2021)]. Outside the clinical applications, AOT appears promising also in promoting motor learning, as witnessed by studies investigating its contribution to the acquisition of new motor skills in both generic upper limb tasks (Shea et al., 2000; Andrieux and Proteau, 2013; Simones et al., 2017; Larssen et al., 2021) as well as sports (Weeks and Anderson, 2000; Al-Abood et al., 2010; Kim et al., 2010; Bouazizi et al., 2014; Sakadjian et al., 2014).

The main aim of the present study was (1) to evaluate the efficacy of AOT in sustaining the learning of new complex motor skills and (2) to compare its efficacy against observational learning and motor practice. We enrolled 54 healthy naive volunteers administering AOT, observational learning, or motor practice training to promote knot tying learning. Contrary to most of the previous AOT literature, we designed the three motor learning procedures balanced in terms of stimulation of the motor system, i.e., containing the same number of blocks regardless of their executional or observational nature. If one between action observation and execution has a prominent role in favoring motor learning, observational learning or motor practice should have a behavioral advantage over the other training procedure, and AOT should exhibit an intermediate efficacy. In a more complex view, however, not only the individual elements but also their combination in a given temporal sequence would contribute to the motor training outcome. If the regular alternation between action observation and execution is an added value in driving motor learning, subjects undergoing AOT should present the highest learning rates. Whatever the results, the present study would contribute to the definition of motor learning optimization procedures and establish the AOT usability in acquiring several motor skills required in routine activities.

## Materials and Methods

**FIGURE 1:**
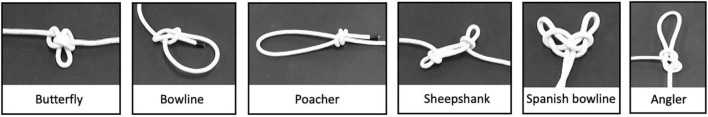
Experimental stimuli. Final configuration of the six nautical knots.

The present study aims at evaluating the efficacy of different training procedures in promoting the learning of new, complex motor skills. We selected six different nautical knots, as a previous study involving tying knots proved that both motor practice and observational learning activated the fronto-parietal networks (Cross et al., 2017). Six knots (see Figure 1) were selected among the most common nautical knots, ensuring that they required the use of a single rope without any additional tool.

An online questionnaire was first administered to 49 volunteers (age *M* = 27.89, *SD* = 4.06, 34 females) who reported no previous knots experience (e.g., fishing, sailing, and climbing). Participants were asked to observe videos (lasting 22–36 s) (see Supplementary Videos) showing an expert performing the knots from an egocentric perspective and estimate the number of observations ideally needed to enable them to reproduce each of the knots correctly. They were instructed to observe each video only once before answering. Subjects indicated, on average, that 5.25 observations would have allowed them to successfully replicate all the knots (see Supplementary Material, Section Questionnaire Results). This information was then used to design the subsequent experimental phase, particularly the AOT training.

### Participants

Fifty-four healthy naïve volunteers (age *M* = 26.15, *SD* = 3.87, 35 females) were subsequently enrolled for the behavioral experiment. None of them had been previously recruited for the online questionnaire. According to the Edinburgh Handedness Inventory (Oldfield, 1971), all participants were right-handed (M = 0.76, SD = 0.15), had a normal or corrected-to-normal vision and no history of neurological or psychiatric disorder.

We conducted an *a priori* power analysis for within/between-subjects ANOVA with G-Power 3.1 to define the sample size suitable for our study. The analysis output showed a minimum sample size of 45 subjects (15 for each group) to obtain a significant effect on the dependent variable with an α = 0.05, a power β = 0.90, and a large effect size (Cohen’s F = 0.4). Our recruitment plan envisioned an excess of 20% of subjects, i.e., increasing from 45 to 54, to limit the consequences of possible drop-outs or participants meeting the exclusion criteria for the neuropsychological battery.

The group assignment was performed via simple randomization, i.e., each participant was assigned to one of the three groups purely randomly for every assignment via a computer-generated label. As such a method has no memory, i.e., it does not account for previous assignments, there is no guarantee that the final sampling is perfectly balanced. The experimental conditions were: Action Observation Training (AOT, *n* = 22; age M = 26.14, *SD* = 3.91), Observational Learning (OL, *n* = 16; age *M* = 25.75, *SD* = 4.55), and Motor Practice (MP, *n* = 16; age *M* = 26.56, *SD* = 3.27).

The study was approved by the local ethics committee (Comitato Etico dell’Area Vasta Emilia Nord, n. 10084, 12.03.2018) and was conducted according to the principles expressed in the Declaration of Helsinki. The participants provided written informed consent.

### Neuropsychological Assessment

Cognitive level and visuospatial working memory measures were collected at baseline to verify the absence of cognitive and memory impairments. The cognitive level was assessed with the Raven’s Standard Progressive Matrices (SPM Raven, 2008); this test measures the ability to extrapolate a rule from a visuospatial configuration of elements and utilize it to identify the correct element to complete the configuration (Alderton and Larson, 1990). A cut-off was set at the 25th percentile [Raven (2008), SPM Manual]. A digital version (PEBL Mueller and Piper, 2014) of Corsi Block Span (Corsi, 1972; Kessels et al., 2000) was used to evaluate the visuospatial working memory (i.e., the capacity to reproduce sequences of blocks of increasing length after their demonstration). A cut-off was calculated based on normative data [average ± *SD*: 6.2 ± 1.3, see Kessels et al. (2000)]. Subjects with scores lower than 3.6 (i.e., <2 standard deviations from the normative mean) were excluded.

As knot tying learning may be affected by individual visuospatial and imitation skills, all participants underwent a neuropsychological assessment of these abilities before the experiment. The Surface Development Test (SDT) (Ekstrom et al., 1976) was selected to measure the ability of spatial visualization (Linn and Petersen, 1985). The test investigates the capacity to manipulate a 3D object mentally and analyze the relationship between its different parts. The imitation skills were assessed with an adapted version of the Manual Motor Sequences test (Nepsy II; Korkman et al., 2011). Participants were asked to observe and then replicate bimanual motor sequences executed by an actor. The detailed description of the tests and baseline scores is reported in the Supplementary Material (Section Neuropsychological Assessment).

### Stimuli and Experimental Design

The stimuli consisted of videos depicting the execution of six nautical knots (see Figure 1). An expert executed all knots with a rope (Length 1 m, Diameter 8 mm) and her performance was video recorded from an egocentric perspective. In the attempt to decompose the knot tying action, we segmented each knot tying in consecutive steps, each reflecting a switch in the configuration of the rope.

Participants sat comfortably at a table, with hands resting in an initial position. The stimuli were presented using PsychToolbox-3 (Brainard, 1997; Pelli, 1997; Kleiner et al., 2007) on a monitor (22 inch LCD) placed at 60 cm from the participant’s forehead. A white rope was horizontally positioned at a distance of 25 cm from the edge of the table, lying on a tape strip.

**FIGURE 2:**
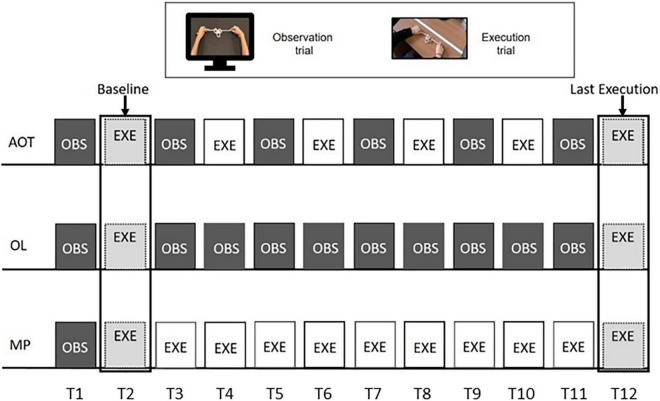
Experimental design. The three rows represent the procedures of action observation training (AOT) (top), observational learning (OL) (middle), and motor practice (MP) (bottom) training. All of them are based on 12 trials, based on action observation (dark gray color) or action execution (white). Light gray indicates the execution trials common to all groups.

Subjects belonging to the AOT group were administered with alternated observation and execution trials (see Figure 2, upper string). Twelve trials were chosen to meet the six observation trials suggested by the online questionnaire results. During the observation trial (lasting 22–36 s), subjects were asked to observe the expert performing the knot without making any movements with their hands. Then, participants had to execute the knot they had just seen. The execution trial lasted twice as long as the observation one (thus lasting 44–72 s).

The observational learning (OL) group started the experiment with an observation-execution block. Subsequently, participants kept observing the knot videos until the final execution (see Figure 2, middle string). Likewise, after the initial observation-execution block, MP participants were requested to practice motorically until the end of the training (see Figure 2, lower string). As the MP training lasted about 10 min for each knot and participants could lose focus about the goal to achieve, a static image of the final knot configuration was presented for 5 s before each MP execution trial. However, neither dynamical stimuli nor other cues about the movements-to-execute were presented.

Two acoustic signals indicated the start and end of each execution trial, during which participants were free to attempt the knot tying with no feedback from the experimenter. Therefore, subjects could make multiple attempts within each execution trial, as they could realize to be on the wrong track and, for this reason, restart from scratch. As detailed below, all attempts were considered in the analysis (see Supplementary Material, Section Number of Attempts for Each Knot and Condition, for the average number of attempts per knot and group). We discarded only the attempts that timed out, provided they were started with not enough time for the knot to be accomplished. Each participant’s performance was video-recorded, and the scoring was performed offline by two experimenters. Each participant repeated the specific training for the six knots, whose presentation order was randomized. Despite the different procedures required by AOT, OL, and MP training, it is worth noting that all three envision the same number of trials, ideally balancing the amount of stimulation delivered to the participant motor system. In addition, only the first (OBS), second (EXE), and last (EXE) trials are common for all three groups. As indicated in Figure 2, the first execution was considered a baseline, while the last one was used to quantify the performance increase for each subject relative to the baseline.

### Data Scoring

For each attempt, the individual performance was scored using a binary index of success, where “1” and “0” were respectively attributed in case of successful execution or incorrect/incomplete knot tying.

We computed the success rate for each trial and knot, averaging the success indices across all the attempts. We discarded only the attempts which timed out, provided that they were started with not enough time for the subjects to accomplish the knot (e.g., for a knot whose video lasts 22 s, a timed out attempt lasting 25 s was counted as a failure, another one lasting 10 s was not considered). Subsequently, we averaged the Success Rate scores across the knots for each execution trial. Therefore, we obtained six scores for the AOT group, two for OL, and eleven for the MP group (see EXE blocks in Figure 2).

Although the success rate is an objective measurement of motor learning, one may argue that it might be too rigid, lacking to reveal partial motor performance improvement. To overcome this issue, we calculated two additional parameters complementing the success rate but more sensitive to the subtle yet meaningful improvement of the knot tying performance. The first was the Correct Steps Rate, i.e., the percentage of steps correctly performed within each knot tying attempt by the participants (for more details, see Supplementary Material, Section Performance Improvement in Terms of Correct Steps). Second, by computing the time-to-completion of successful attempts, we measured the speeding up of MP and AOT participants over the training, proposing this as a further index of partial motor improvement (see Supplementary Material, Section Evaluation of the Time-to-Completion).

### Statistical Analysis

Neuropsychological scores and age were submitted to a Shapiro-Wilks W-Test to verify the assumption of normality. Parametric (one-way ANOVA, Bonferroni *post hoc*) or non-parametric tests (Kruskal-Wallis, Mann-Whitney *post hoc*) were applied accordingly to ascertain that the three groups were balanced. The group balance was further verified on baseline performance via the Kruskal-Wallis test. In addition, Bayesian statistics was implemented to index the probability that our three groups were balanced in terms of neuropsychological scores, age, and baseline performance. The Bayes factor was computed to express how many times H0 is more likely to occur than H1 (BF_01_). The BF robustness check indicates the level of evidence (Jeffreys, 1998; Jarosz and Wiley, 2014).

We applied a Wilcoxon test to compare the success rates of the first and last execution trials and verify whether all training had induced a significant performance improvement along the training period. To compare the efficacy of the three training, we then computed the performance improvement score as the success rate of the final execution minus the baseline score and then compared these scores across groups with Kruskal-Wallis and Mann-Whitney tests. Eta-squared (η^2^) was calculated as a measure of effect size. The same analyses were conducted on the correct steps rate. The Bayesian statistics (BF_10_) was calculated to measure the probability of the real contribution of the training on the performance improvement (i.e., how many times H1 is more likely to occur than H0, BF_10_).

As the performance improvement reflects only an overall picture of the training efficacy, also intermediate scores were baseline-corrected and compared when available (i.e., AOT vs. MP at four intermediate time points) via a Mann-Whitney test.

Beyond the population level, we conducted two analyses to test the relationship between individual neuropsychological scores and performance improvement. First, a non-parametric correlation (Spearman) was tested between the performance improvement scores and visuospatial and imitation abilities. Second, a multiple regression was applied within each group to investigate whether visuospatial and imitation scores could predict the learning outcome of MP, OL, and AOT groups.

## Results

All the 54 participants showed neuropsychological scores above the cut-off values, so none of the enrolled subjects was excluded. In particular, Raven’s SPM scores all exceeded the 64th percentile, while all Corsi Block Span scores were ≥5.

Due to the absence of normality assumption, all the neuropsychological variables underwent non-parametric analyses. The Kruskal-Wallis test returned no significant main effect of group for any neuropsychological scores (all *p* > 0.38). Also the age of participants was not significantly different across groups [*F*_(2,51)_ = 0.17, *p* = 0.84]. At baseline, the three groups exhibited very similar initial levels (around 10% of success rate) with no significant group effect [*H*_(2,54)_ = 0.43, *p* = 0.81]. The Bayes Factors (BF_01_) confirmed all these observations, indicating at least substantial evidence for the null hypothesis for all the variables (all BF_01_ > 3.20).

**FIGURE 3:**
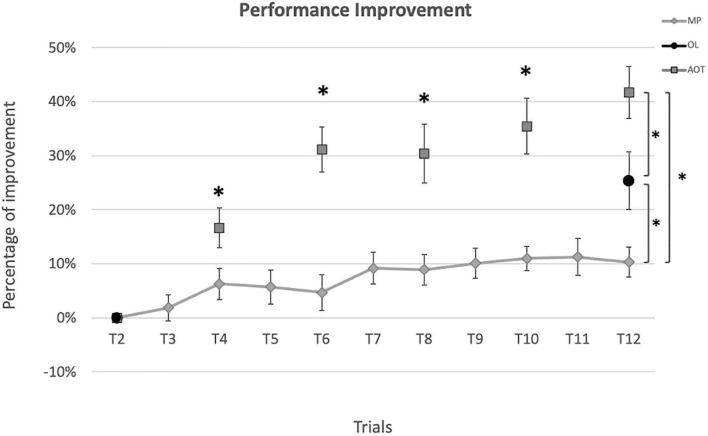
Performance improvement. Performance improvement (%) evaluated at each execution trial relative to the baseline. Diamonds represent the motor practice (MP) scores, circles represent the observational learning (OL) scores, and squares represent the action observation training (AOT) scores. Asterisks indicate significant differences at the Mann-Whitney test (*p* < 0.05).

All groups exhibited a significant learning effect along the training (see Figure 3), as demonstrated by the significantly better performance at the end of the training compared to baseline (MP improvement: 10%, Wilcoxon: Z = 2.76, *p* = 0.006; OL improvement: 25%, Wilcoxon: Z = 3.20, *p* < 0.001; AOT improvement: 42%, Wilcoxon: Z = 4.07, *p* < 0.001). The Kruskal-Wallis test conducted on the performance improvement score (i.e., the global before-after training improvement) showed a significant effect of group [*H*_(2,54)_ = 19.35, *p* < 0.001, η^2^ = 0.34, BF_10_ = 536.33]. *Post hoc* comparisons revealed that AOT subjects outperformed relative to both MP and OL participants (*p* < 0.001 and *p* = 0.03 respectively), with these latter showing significantly better scores than MP (*p* = 0.03). Similar findings were obtained by applying bootstrap statistics to overcome the uneven sampling of our groups (see Supplementary Materials, Section Bootstrap Permutation).

To explore the trial-by-trial dynamics of improvement induced by AOT, we further conducted Mann-Whitney tests between MP and AOT performance improvement at intermediate and common time points (i.e., T4 – T6 – T8 – T10). A significant difference between the two groups emerged since the fourth trial and was maintained over time (T4: *p* = 0.45; T6: *p* < 0.001; T8: *p* = 0.001; T10: *p* < 0.001).

The analyses of the correct steps rate fully confirm the results relative to the success rate (see Supplementary Material, Section Performance Improvement in Terms of Correct Steps). In addition, also the evaluation of the time-to-completion revealed that the effects of motor learning are not limited to the increase of success rate and correct steps but extend to the speed of the participants in knot tying, with an average time decrease for AOT and MP participants around the 25–30% (for more details see Supplementary Material, Section Evaluation of the Time-to-Completion). Since OL participants attempt only twice the knot execution, the time-to-completion decrease can be computed only in participants succeeding since the very first trial, and this explain their sparse and less stable results.

The correlation analysis showed that both visuospatial abilities and imitation skills positively and significantly correlate with the performance improvement in the AOT group (ρs = 0.65, *p* < 0.001 and ρs = 0.46, *p* = 0.03, respectively). In contrast, neither visuospatial abilities nor imitation skills are correlated with the performance improvement in the MP group (ρs = 0.18, *p* = 0.49 and ρs = –0.07 *p* = 0.78, respectively). Moving to the OL group, an intermediate pattern emerges, whereby performance improvement correlates only with the imitation skills (ρs = 0.62, *p* = 0.009), but not with visuospatial abilities (ρs = 0.39 *p* = 0.13) (see Supplementary Materials, Section Correlational Analysis).

Moving to the multiple regressions, an overall pattern similar to that depicted from the correlation analyses emerges. The model results not significant for the MP group (LearningRate = 0.13 + 0.39*SDTscore–0.37*MMSscore; *p* = 0.4, R^2^ = 0.10). Concerning the OL and AOT groups, the models are significant, explaining the 40% and the 51% of the learning rate variance, respectively (OL: LearningRate = -0.07 + 0.08*SDTscore + 0.59*MMSscore; *p* = 0.03, R^2^ = 0.40; AOT: LearningRate = -0.09 + 0.51*SDTscore + 0.42*MMSscore; *p* = 0.001, R^2^ = 0.51). A main role of imitation abilities emerges for the OL group, evidencing that individual MMS scores drive most of the learning performance, while SDT scores play a marginal role (βmms = 0.59 vs. βsdt = 0.08). Interestingly, in the AOT group, both visuospatial and imitation abilities were estimated to determine a quote of learning performance, with an effect of 0.51 and 0.42, respectively.

## Discussion

The present study aimed to (1) investigate whether AOT sustains the acquisition of new complex motor skills and (2) compare its efficacy with observational learning or motor practice. To this purpose, 54 naïve healthy subjects were recruited and randomly assigned into three groups (AOT, OL, MP), receiving the same amount of stimulation (12 trials) but with different dosages of action observation and execution.

Action observation and execution elicit shared motor representations in the fronto-parietal circuits in both humans (Rizzolatti et al., 2014; Hardwick et al., 2018) and non-human primates (Nelissen et al., 2011; Bonini, 2017). Interestingly, previous brain imaging findings demonstrated that the activation of the fronto-parietal networks elicited by action observation sets the premises for subsequent imitation learning (Buccino et al., 2004; Iacoboni, 2005; Vogt et al., 2007; Sakreida et al., 2018). Buccino et al. (2004) administered functional MRI to participants while they observed an unknown motor action (playing guitar chords) to be subsequently executed. They highlighted that action observation elicits the motor act representations through the activation of the parietal and frontal lobes, whereas the additional activation of the prefrontal lobe (area 46) during the subsequent motor preparation is required to recombine such motor acts into a new motor program. Such a process is advanced as the neural basis of imitation learning (Buccino et al., 2004). Starting from this premise, not only action execution (Kami et al., 1995; Doyon and Benali, 2005; Dayan and Cohen, 2011; Gryga et al., 2012; Chang, 2014; Lage et al., 2015) but also action observation can favor plastic changes in the cortical motor system (Stefan et al., 2005; McGregor and Gribble, 2015; Lagravinese et al., 2017; McGregor et al., 2018), and thus promote motor learning (Mattar and Gribble, 2005; Stefan et al., 2005, 2008). However, whether a training *regularly* alternating action observation and execution may surpass the pure motor practice and observational learning remains to be established.

The main result of our study revealed a significantly higher performance improvement for the AOT than both OL and MP groups, indicating that the more effective way to promote motor learning is represented by the regular alternation between observation and execution. How can we explain the superiority of AOT training? In motor practice, the subject adjusts and corrects his performance relying only on his feedback (e.g., proprioception, goal achievement), with no external cues driving toward better performance. Observational learning has, in principle, the capacity to overcome this limitation, but it fails to make the subject experience the motor execution, thus lacking any praxic and proprioceptive components. Only AOT, then, combines the two sides of the motor experience. Indeed, the regular alternation between observation and execution makes the first activate the motor system according to the correct motor program, whereas the latter makes the subject execute the action with a motor system already pre-activated and geared toward a correct performance. The reiteration of this sequence provides an incremental, adjunct value that super-adds onto MP and OL efficacy. Indeed, the performance improvement driven by AOT (42%) is larger than the sum of those due to MP and OL (10% + 25% = 35%). One could wonder whether the online combination of observation and execution would serve the training purposes as well. However, the lack of sequentiality could limit the advantages of such a procedure because of the higher cognitive load requested by the simultaneous accomplishment of two processes. Even more, action observation and execution share similar neural substrates, thus some forms of interference cannot be discarded, further impacting the motor learning rate.

Different, not mutually exclusive neurophysiological models can be advanced to explain how AOT can contribute to complex motor learning. The formation of novel motor engrams may be favored by the overlap between the cortical areas activated by action observation and action execution (Hardwick et al., 2018). Especially at the inferior parietal level, previous studies (Fogassi et al., 2005) showed that different motor acts are not represented independently during action execution and observation but are chained together according to the overall action intention. Observing the entire knot tying should have primed a similar neural activity during the subsequent motor execution. In addition, consolidating complex motor skills requires the involvement of brain areas not endowed with mirror mechanisms like the prefrontal cortex and temporo-mesial structures. Here, it must be noted that both these hubs are targeted by dense cortico-cortical projections from parietal areas with mirror properties (Rizzolatti and Luppino, 2001). Thus, action observation could result in the facilitation also of these structures, ultimately supporting the improvement of motor performance. Finally, it is worth mentioning that also extrapyramidal structures like basal-ganglia and cerebellum may have driven, via the mirror mechanism [see Prather et al. (2008); Bonini (2017), and Errante and Fogassi (2020)], the automatization of basic, procedural movements (e.g., loops formations), further supporting the motor performance.

Action-related sensory input not only evokes neural activity in motor pathways but also affects motor behavior. It was suggested that the combination of action observation and physical practice provides more unique opportunities than either observational learning or physical practice alone [e.g., Shea et al. (2000) and Weeks and Anderson (2000)]. Evidence in favor of this conclusion has been provided in a wide range of sports such as soccer (Hodges et al., 2005), cricket (Breslin et al., 2006), bowling (Hayes et al., 2007), weightlifting (Sakadjian et al., 2014), volleyball (Weeks and Anderson, 2000), and golf (Guadagnoli et al., 2002; Kim et al., 2010; D’Innocenzo et al., 2016; Nishizawa and Kimura, 2017). All these studies proved that the combination of OL and MP facilitates the learning of motor skills, especially in terms of changes in performance accuracy and movement kinematics.

Despite the superiority of AOT in driving the acquisition of new motor skills, OL and (to a minor extent) MP succeeded in inducing a performance improvement. Concerning observational learning, it is not surprising that the repeated observation of an expert performing an action represents a good drive for the acquisition of new motor skills [see Ferrari (1996); Horn and Williams (2004), Hodges et al. (2007); Vogt and Thomaschke (2007), Ste-Marie et al. (2012), and Mizuguchi and Kanosue (2017)].

In a series of fMRI studies, Cross et al. (2006, 2009) compared physical practice with observational learning, showing that both have beneficial effects grounding on the activation of premotor and inferior parietal regions. Interestingly, the same group confirmed these results also for a nautical knot learning task (Cross et al., 2017). However, in contrast with our findings, they reported a larger improvement for physical practice relative to observational learning. This discrepancy, however, could be ascribed to the different experimental procedures adopted in the two studies. Indeed, while our MP participants made only one initial observation and then had to keep practicing only motorically, in Cross et al. (2017), participants undergoing physical practice could start, stop and restart the video of the knot tying at any time, thus resembling more to an AOT than a mere MP.

The lowest performance improvement was obtained in the MP group, which was the only one without visual guidance. Then, the mere trial-and-error practice without visual cues (or visual feedback given by the expert observation) prevents, or at least limits, the online adjustments and then determines the lowest performance improvement. Nevertheless, an improvement is still observed (about 10%), and this finding is in line with previous studies on similar tasks. For instance, Tracy et al. (2003) administered motor practice about eight nautical knots and compared the motor performance before and after the training, reporting a significant decrease in the time-to-completion and increase in the proportion of knots tied correctly. Even in this study, however, the motor practice was “contaminated” with visual cues, as subjects had the chance to look at static pictures depicting the final knot configuration and each step of the tying procedure. This aspect could explain why the overall performance improvement observed by Tracy et al. (2003) is higher than the one reported in our MP group (around 49 vs. 10%).

Finally, we conducted a correlational analysis to identify the neuropsychological functions mostly underlying the AOT efficacy and evaluate whether the possible relationships are specific for the AOT training or globally valid for motor learning regardless of the type of training. The performance improvement induced by AOT positively and significantly correlates with both visuospatial abilities and imitation skills. These findings are not surprising, as the first competence is massively required by a sequential, visuospatial task like knot-tying, while the latter ability underlies the capacity to transfer valuable information from action observation to execution. Most interestingly, these results appear quite specific for AOT. Indeed, none of the two variables correlate with the performance improvement of the MP group, while only imitation skills significantly correlate with the outcome of OL. This latter evidence likely reflects the need of participants exposed to visual cues (i.e., AOT and OL, but not MP) to retain the information and deploy it in the execution phase. The higher this capacity in the individual, the higher the training efficacy.

The regression analyses enrich what we showed by correlation and confirm a role of pre-existent imitation abilities in sustaining the learning performance in participants involved in OL and AOT training. Nevertheless, in the specific case of the AOT, the level of visuospatial abilities also sets better premises for motor learning.

A few limitations must be acknowledged in our study. The first is related to the limited numerosity of our sample, which is a key point in studies about learning, often leading to underpowered and overworked results (Lohse et al., 2016). Although our results are not underpowered, extending the investigation to a larger cohort of subjects would reinforce our results, especially those related to regression and outcome prediction. A second aspect concerns a certain “rigidity” inherent to our main outcome, a binary success score. While this is in principle true, an additional analysis addressing the percentage of correct steps performed in each attempt confirmed the previous findings, thus indicating that the rigidity of the success rates poorly affected our conclusions. However, future studies could consider not only the movement outcome as an endpoint but also kinematic measurements providing similarity indexes between the observed model and the trainee (De Marco et al., 2020, 2021). Such indexes would reflect the appraisal of “a motion pattern” more than the mere performance success. In addition, neural indicators could uncover the neurophysiological substrates of AOT efficacy, opening to the adoption of non-invasive brain stimulation techniques to predict or boost its efficacy (Nuara et al., 2021).

Finally, keeping the three training balanced in terms of stimulation of the motor system required us to design procedures that could sound not balanced in terms of difficulty and mnemonic load. However, such aspects cannot explain our results but only account for a limited effect. Indeed, the memory load requested to our participants was highest in MP, intermediate in AOT, and lowest in OL. Thus, while high memory load could have mitigated the motor learning of MP participants, it cannot explain why AOT subjects outperform OL. To limit the impact of the higher mnemonic demand requested to the MP participants, we administered our participants with a static image depicting the final knot configuration. Concerning the amount of exposure to the actor performance, although the OL group received more observation trials than the AOT group, the performance improvement achieved by the OL participants did not reach the improvement shown by AOT participants. Thus, the alternation of the two elements, rather than the amount of exposure to the observational stimuli, seems to sustain the performance improvement. In our experimental design, we adopted a regular alternation of the two conditions. Whether an irregular but still alternated sequence can induce motor learning comparable with a regular pattern needs to be investigated by future studies, but the underlying neurophysiological models make us suppose suboptimal training outcomes.

In conclusion, our results demonstrated that the regular alternation of action observation and execution (namely, AOT) might generate a synergistic effect leading to a super-additive efficacy in acquiring new motor skills. In other words, the performance improvement warranted by AOT is higher than the sum of those achieved via motor practice and observational learning. This effect relies on the capacity of action observation to activate the cortical motor system, tuning the formation of new motor programs. Several possibilities stem from these findings, extending the use of AOT from the clinical, rehabilitative context to daily routines requiring the learning and perfectioning of new motor skills. Seminal examples are sports training, music, and occupational activities requiring fine motor control.

## Data Availability Statement

The raw data supporting the conclusions of this article will be made available by the authors, without undue reservation.

## Ethics Statement

The studies involving human participants were reviewed and approved by Comitato Etico dell’Area Vasta Emilia Nord, prot. n. 10084, 12.03.2018. The patients/participants provided their written informed consent to participate in this study.

## Author Contributions

MB designed the study, performed the research, analyzed data, and wrote the manuscript. AN designed the study, analyzed data, and wrote the manuscript. ES performed the research. DD analyzed data. GR wrote the manuscript. PA and MF-D designed the study and wrote the manuscript. All authors contributed to the article and approved the submitted version.

## Conflict of Interest

The authors declare that the research was conducted in the absence of any commercial or financial relationships that could be construed as a potential conflict of interest.

## Publisher’s Note

All claims expressed in this article are solely those of the authors and do not necessarily represent those of their affiliated organizations, or those of the publisher, the editors and the reviewers. Any product that may be evaluated in this article, or claim that may be made by its manufacturer, is not guaranteed or endorsed by the publisher.
