# Supplementary material for: The Proactive Synergy Between Action Observation and Execution in the Acquisition of New Motor Skills
Source: Front Hum Neurosci. 2022 Mar 24;16:793849. doi: 10.3389/fnhum.2022.793849 (PMC8986982; doi:10.3389/fnhum.2022.793849)
Supplement: Supplementary file 2 [file Data_Sheet_2.docx]

**The proactive synergy between action observation and execution in the acquisition of new motor skills**

Maria Chiara Bazzini^1,2^, Arturo Nuara^1^, Emilia Scalona^1^, Doriana De Marco^1^, Giacomo Rizzolatti^1,2^, Pietro Avanzini^1,3^, Maddalena Fabbri-Destro^1*^

^1^Consiglio Nazionale delle Ricerche, Istituto di Neuroscienze, Parma, Italy.

^2^Università degli Studi di Parma, Dipartimento di Medicina e Chirurgia, Parma, Italy.

^3^Istituto Clinico Humanitas, Humanitas Clinical and Research Center, Rozzano, Milano, Italy.

*** Correspondence:**

Maddalena Fabbri-Destro

e-mail: [maddalena.fabbridestro@in.cnr.it](mailto:maddalena.fabbridestro@in.cnr.it)

***Supplementary Material***

**Section 1. Questionnaire results**

|  | Butterfly | Bowline | Poacher | Sheepshank | Spanish Bowline | Angler |
| --- | --- | --- | --- | --- | --- | --- |
| Number of Observations | *M* = 3.24  *SD* = 1.26 | *M* = 2.99  *SD* = 1.45 | *M* = 2.39  *SD* = 1.38 | *M* = 4.02  *SD* = 1.99 | *M* = 5.25  *SD* = 2.44 | *M* = 4.52  *SD* = 1.86 |

**Supplementary Table 1.** **Questionnaire results.** Mean and standard deviation of the number of observations ideally enabling participants to reproduce each knot correctly.

**Section 2. Neuropsychological assessment**

The Raven's Standard Progressive Matrices (SPM Raven, 2008) and the Corsi Block Span (Corsi, 1972; Kessels et al., 2000) (digital version PEBL Mueller and Piper, 2014) were used to evaluate the cognitive level and the visuospatial working memory.

The Surface Development Test (Ekstrom et al., 1976) was administered to evaluate the visuospatial abilities. The test comprises two parts with 30 points as the maximum score for each part. The sum of the two scores, thus ranging between 0 and 60, reflects the total score.

The imitation abilities were assessed using an adapted version of the subtest of Nepsy II, namely Manual Motor Sequences. The test was composed of 8 bimanual motor sequences in which the participants had to replicate each sequence six times immediately after observing the actor's performance. Participants' execution was video recorded and scored offline, attributing a score of 1 to each sequence correctly performed and 0 to those wrongly executed. The sum of the corrected sequences constitutes the total score obtained by the subject (ranging between 0-48)

|  | MP | OL | AOT |
| --- | --- | --- | --- |
| Age | *M* = 26.14 *SD* = 3.91 | *M* = 25.75 *SD* = 4.55 | *M* = 26.56 *SD* = 3.27 |
| SPM Percentile | *M* = 93.56 *SD* = 8.84 | *M* = 93.56 *SD* = 8.80 | *M* = 93.50 *SD* = 7.61 |
| Corsi Block Span | *M* = 6.00 *SD* = 0.73 | *M* = 6.06 *SD* = 0.77 | *M* = 5.91 *SD* = 1.19 |
| SDT | *M* = 35.31 *SD* = 15.28 | *M* = 32.50 *SD* = 13.48 | *M* = 34.09 *SD* = 15.04 |
| MMS test | *M* = 22.00 *SD* = 7.32 | *M* = 18.00 *SD* = 7.98 | *M* = 20.00 *SD* = 7.75 |
| Success Rate Baseline | *M* = 0.10 *SE* = 0.03 | *M* = 0.11 *SE* = 0.03 | *M* = 0.10 *SE* = 0.03 |

**Supplementary Table 2.** **Baseline scores.** Means and dispersion indices (standard deviation or standard error) of age, neuropsychological scores (Raven's SPM, Corsi Block Span, Surface Development Test, and adapted Manual Motor Sequences), and success rate at baseline for the three groups (MP, OL, and AOT).

**Section 3. Number of attempts for each knot and condition**

During each execution trial, participants were free to attempt the knot tying with no feedback from the experimenter. Therefore, subjects could make multiple attempts within each execution trial, as they could realize to be on the wrong track and, for this reason, restart from scratch. All attempts were considered in the analysis, provided they were started with enough time for the knot to be accomplished.

The average number of attempts for each knot and group is reported in the Table below.

|  | ***Mean number of attempts MP*** | ***Mean number of attempts OL*** | ***Mean number of attempts AOT*** |
| --- | --- | --- | --- |
| ***Butterfly*** | 1.34 | 1.53 | 1.67 |
| ***Bowline*** | 1.34 | 1.33 | 1.55 |
| ***Poacher*** | 1.24 | 1.20 | 1.45 |
| ***Sheepshank*** | 1.45 | 1.43 | 1.64 |
| ***Spanish bowline*** | 1.47 | 1.43 | 1.73 |
| ***Angler*** | 1.40 | 1.37 | 1.66 |

**Supplementary Table 3.** **Average number of attempts for each knot and group.**

**Section 4. Performance improvement in terms of Correct Steps**

In the results section, we presented the success rate of our participants as the main outcome of the three motor learning procedures. However, one could argue that such a measurement is too rigid, making the outcome of the motor performance binary and thus devoid of any slight, suboptimal yet meaningful degree of learning. To overcome this issue, we took advantage of the sequential nature of our task, complementing our findings by computing the number of steps correctly performed within each knot tying attempt. Indeed, even in the case of a systematically failed knot performance, subjects could increase the number of steps correctly reproduced. For example, for a knot including six steps, one could make only one correct step in trial one and reach four correct steps in trial six. In this case, the success rate would remain stable to 0%, but the rate of correct steps would increase from 16% to 66%, thus indicating the achievement of partial success. As a note, we opted for the percentage of correct steps and not their absolute number because the 6 knots were composed by a different number of steps (Butterfly: 4 steps; Bowline: 4 steps; Poacher: 5 steps; Sheepshank: 5 steps; Spanish bowline: 6 steps; Angler: 6 steps). If a trial contained multiple attempts, we averaged the correct steps rate. Finally, the correct step rate was averaged across the knots.

To evaluate the balance of the three groups in terms of initial performance, we conducted a Kruskal Wallis test on the baseline scores. The three groups exhibited very similar initial scores (AOT: mean = 0.37, es = 0.03 MP: mean = 0.38, es = 0.04; OL: mean = 0.33, es =0.02), with no significant difference among groups (H(2,54) = 1,77, p = 0.41). We used the Wilcoxon test to verify whether all training had induced a significant performance improvement along the training period. All the three groups reached a significant improvement, with the final performance scores significantly higher than baseline for all the groups (MP: Z = 2.84, p = 0.004; OL: Z = 3.51, p < 0.001; AOT: Z = 4.10, p < 0.001) (see Supplementary Figure 1).

Finally, to compare the efficacy of the three treatments, we calculated the improvement in the correct steps rate by subtracting the baseline score of each subject from his/her final performance. The Kruskal-Wallis test conducted on this variable returned a significant effect of group (H(2, 54) = 26,27, p < 0.001, η2 = 0.48). Post hoc comparisons (Mann-Whitney test) showed that the performance improvement in the MP group was significantly lower than in both AOT and OL groups (p < 0.001 and p < 0.001). The performance improvement of the AOT group was the highest, but the difference between AOT and OL did not reach the level of significance (p = 0.27).

As one can see from Supplementary Figure 1, the findings relative to the correct steps rate are almost identical to those presented in the main text concerning the success rate. Indeed, all groups exhibited a significant improvement over time, but AOT subjects outperformed relative to both OL and MP participants, with the latter presenting the lowest improvement. Of note, even the degree of improvement seems quite parallel to that exhibited in success rate. AOT participants improve by 40%, MP by 10%. Both these values are essentially the same visible in Figure 3. Concerning the OL participants, the correct steps rate seems slightly higher than the success rate increase (34% vs. 26%), which also might explain the lack of significance in the post-hoc contrast between AOT and OL.


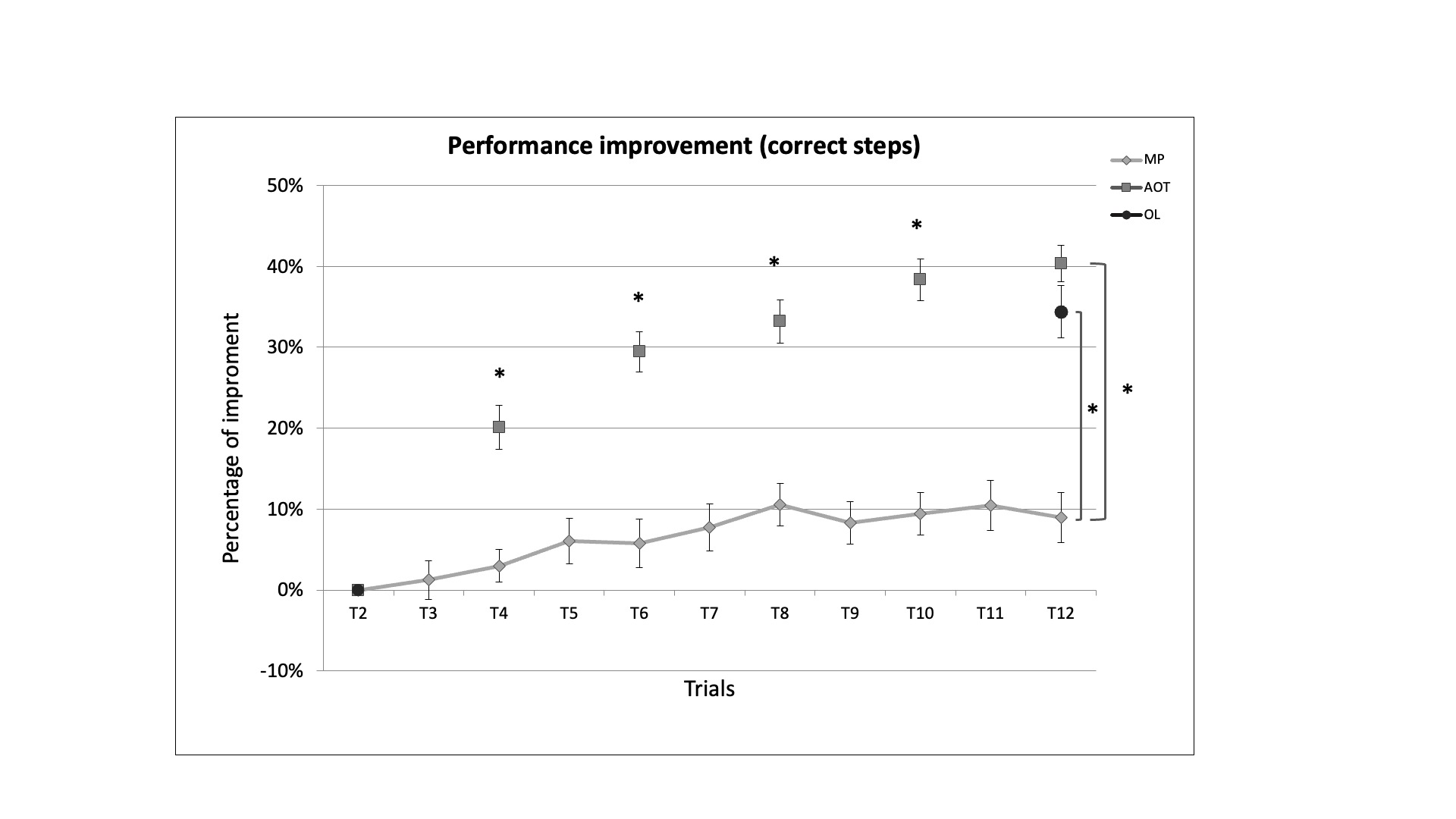


**Supplementary Figure 1**. Performance improvement. Performance improvement (%) evaluated at each execution trial relative to the baseline. Diamonds represent the MP scores, circles represent the OL scores, and squares represent the AOT scores. Asterisks indicate significant differences at the Mann-Whitney test (p < 0.05).

**Section 5. Evaluation of the time-to-completion**

In the results section, we evaluated the success rate to investigate the efficacy of the three training; however, another way to evaluate the degree of learning could be derived from the analysis of the time-to-completion. Once the knot tying procedure is acquired, one might expect that the execution time tends to decrease over time.

Starting from these premises, we computed the time-to-completion for each knot and participant, considering only the successful attempts, i.e., the knots correctly implemented at least twice by each participant along with the training. We computed how much the (correct) knot performance was faster between the first and the last successful execution, considering this as another index of motor learning. The tables below summarize these findings for all the groups. As one can see, the effects of motor learning are not limited to the increase of success rate but also extend to the participants' speed in knot tying, with an average decrease of the time-to-completion around 25-30% for the AOT and MP groups. It is important to underline here that a direct comparison between the two groups would not be fair, as it has been possible to compute the time reduction only on a subset of participants for each knot. For this reason, these data are intended only to demonstrate the presence of learning effects impacting the time of completion, thus going beyond the sole success rate in knots execution. As far as the OL group is concerned, the results appear much less stable. This is not surprising, especially if one considers that they attempt only twice the knot execution, thus the time-to-completion decrease can be computed only in participants succeeding since the very first trial.

| ***AOT group*** | ***N of subjects*** | ***InitialExeTime (s)*** | ***FinalExeTime (s)*** | ***Time decrease***  ***(s)*** | ***%*** |
| --- | --- | --- | --- | --- | --- |
| *Butterfly* | *17* | *23.82* | *19.37* | *-4.45* | *21.11%* |
| *Bowline* | *13* | *23.87* | *19.14* | *-4.73* | *23.54%* |
| *Poacher* | *22* | *24.48* | *19.27* | *-5.21* | *24.24%* |
| *Sheepshank* | *8* | *37.71* | *28.31* | *-9.40* | *29.77%* |
| *Spanish bowline* | *4* | *45.04* | *28.92* | *-16.13* | *49.13%* |
| *Angler* | *6* | *25.60* | *21.07* | *-4.53* | *18.49%* |

**Supplementary Table 4**. Time decreases for the AOT group. For each knot, we reported how many participants succeeded in at least two execution trials, the mean execution time (s) of the first success, the mean execution time of the last success, and the amount of time decrease (seconds and percentage) between the first and the last successful executions.

| ***MP group*** | ***N of subjects*** | ***InitialExeTime (s)*** | ***FinalExeTime (s)*** | ***Time decrease***  ***(s)*** | ***%*** |
| --- | --- | --- | --- | --- | --- |
| *Butterfly* | *4* | *34.06* | *21.46* | *-12.61* | *49.39%* |
| *Bowline* | *1* | *25.40* | *20.56* | *-4.84* | *20.01%* |
| *Poacher* | *14* | *30.78* | *22.63* | *-8.15* | *33.05%* |
| *Sheepshank* | *4* | *35.11* | *17.51* | *-17.60* | *74.19%* |
| *Spanish bowline* | *0* | *N/A* | *N/A* | *N/A* | *N/A* |
| *Angler* | *1* | *33.74* | *21.96* | *-11.79* | *38.80%* |

**Supplementary Table 5**. Time decreases for the MP group. For each knot, we reported how many participants succeeded in at least two execution trials, the mean execution time (s) of the first success, the mean execution time of the last success, and the amount of time decrease (seconds and percentage) between the first and the last successful executions.

| ***OL group*** | ***N of subjects*** | ***InitialExeTime (s)*** | ***FinalExeTime (s)*** | ***Time decrease***  ***(s)*** | ***%*** |
| --- | --- | --- | --- | --- | --- |
| *Butterfly* | *1* | *21.09* | *21.95* | *0.86* | *-4.09%* |
| *Bowline* | *0* | *N/A* | *N/A* | *N/A* | *N/A* |
| *Poacher* | *9* | *30.32* | *30.20* | *-0.12* | *0.39%* |
| *Sheepshank* | *1* | *26.04* | *19.19* | *-6.85* | *26.31%* |
| *Spanish bowline* | *0* | *N/A* | *N/A* | *N/A* | *N/A* |
| *Angler* | *0* | *N/A* | *N/A* | *N/A* | *N/A* |

**Supplementary Table 6**. Time decreases for the OL group. For each knot, we reported how many participants succeeded in at least two execution trials, the mean execution time (s) of the first success, the mean execution time of the last success, and the amount of time decrease (seconds and percentage) between the first and the last successful executions.

**Section 6. Bootstrap permutation**

The simple randomization adopted in our recruitment procedures led to an uneven sampling of our groups. To demonstrate that this aspect did not affect our results, we conducted an additional analysis simulating a sample balanced for the number of subjects among the groups. The index included in the simulation was the performance improvement score of each subject, and 1000 different combinations of twelve subjects from each group were computed, obtaining the average score for each combination.

We selected a numerosity of 12 because it was the maximum number allowing to obtain 1000 independent combinations out of 16 participants. Each group finally contained 1000 mean scores.

We then performed unpaired bootstrapping non-parametric statistics using the "statcond" function provided with the EEGLAB toolbox. The results paralleled those obtained with the original samples, with MP achieving an increment of success rate equal to 7%, OL 20% and AOT 39%. The simulation results confirmed those reported in the study, indicating a significant effect of the group factor (p<0.001).

Finally, a paired non-parametric bootstrap statistical analysis was applied to verify whether the groups significantly differed. The results revealed a significant difference between the AOT and both OL (p<0.001) and MP (p<0.001) groups with the highest performance improvement for the AOT. The OL improvement was significantly greater than the MP group in the OL group (p<0.001).

**Section 7. Correlational analysis**

**
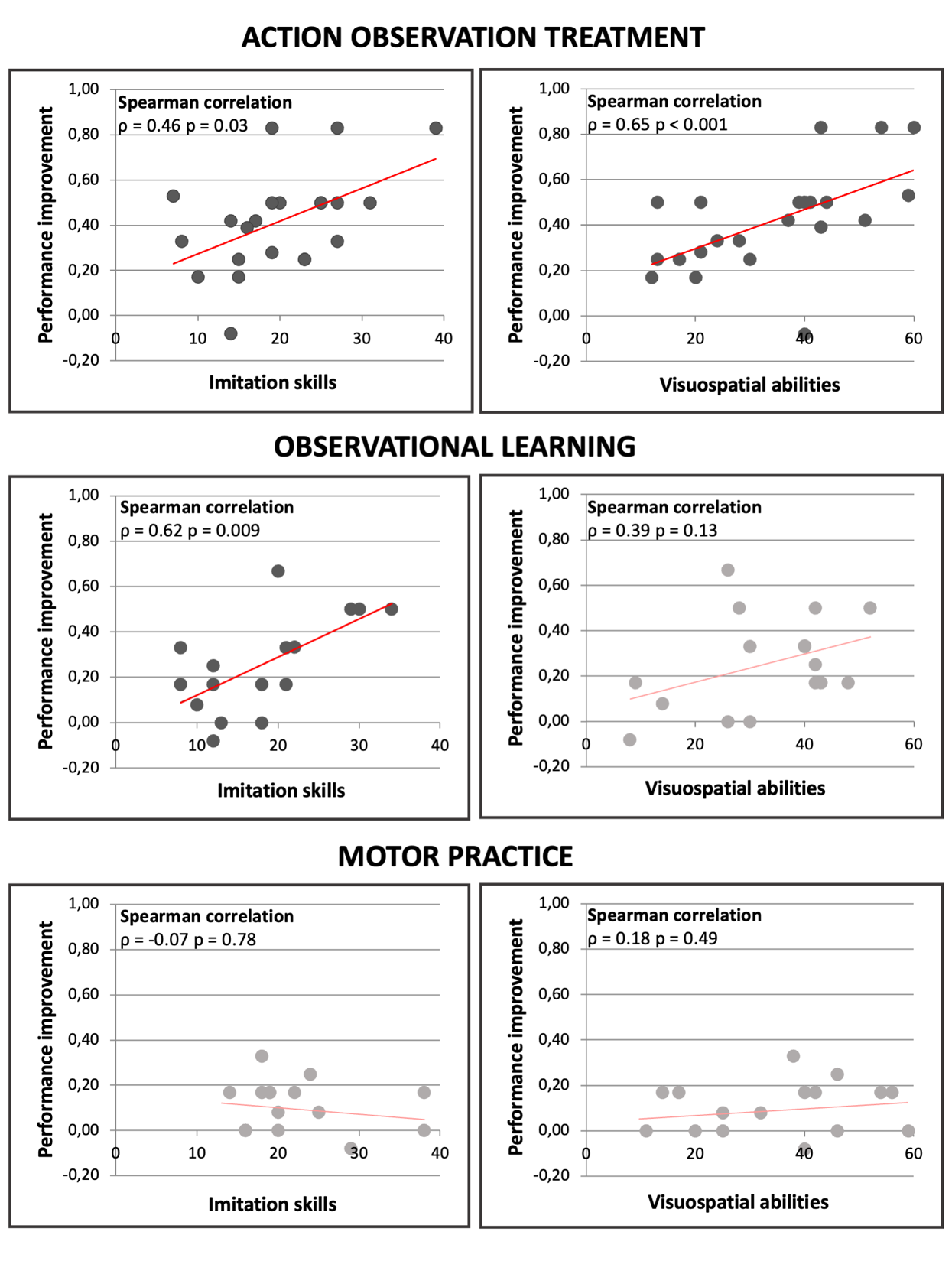
**

**Supplementary Figure 2. Correlational analysis.** Correlation between performance improvement and visuospatial abilities or imitation skills for the three groups (upper AOT; middle OL; lower MP). The red line indicates the linear trend between the two variables, and its thickness reflects the significance of the Spearman correlations, whose results are indicated in the top part of each panel.
